# The challenges of expanding medical student numbers in the UK: A scoping review

**DOI:** 10.1016/j.fhj.2025.100278

**Published:** 2025-06-28

**Authors:** Eli C. Sassoon, Rhiannon C. Craig, David G. O’Brien

**Affiliations:** Education Centre, School of Medicine, The University of Nottingham, Nottingham, UK

**Keywords:** NHS long term workforce plan, Workforce planning, NHS workforce, Undergraduate medical education

## Abstract

**Background:**

With one of the lowest numbers of doctors per capita in Europe, coupled with an ageing UK population, the NHS faces an unprecedented workforce crisis. The NHS Long Term Workforce Plan (LTWP) outlined how this will be addressed, headlining a proposed doubling of UK medical school places.

**Methods:**

A scoping review utilising the Arksey and O’Malley model searched articles related to increasing UK medical student numbers. A total of 6,217 articles were identified, and 23 were selected for further analysis.

**Results:**

Funding was the most frequently raised concern, followed by educator shortage, limited stakeholder engagement, placement capacity shortage, and failure to support Widening Access agendas. Notably, seven articles were opinion pieces, with only two formal research papers.

**Conclusion:**

Key issues to implementing the current LTWP were identified, and further research to address these challenges and identify potential solutions is urgently required to support evidence-based decision making to influence future workforce plans.

## Highlights

• The NHS Long Term Workforce Plan was published in June 2023 by NHS England as its proposal to tackle the NHS workforce crisis.
• The plan included a pledge to double the number of medical students and introduced accelerated undergraduate medical degrees.
• There have been numerous challenges raised with implementation of the plan, including: funding, placement capacity and stakeholder engagement.
• These challenges need to be addressed in the plan’s refresh,due to be published in late 2025.
• The medical profession needs to begin undertaking research to provide evidence to support decision makers involved in NHS workforce planning.

## Introduction

The UK population is ageing, with those aged 85 years or older expected to rise from 1.6 to 2.6 million over the next 15 years, placing unprecedented demands on healthcare provision.^1^ Simultaneously, the NHS is facing a staffing crisis. With a 6.9% vacancy rate and only 3.2 doctors per 1,000 people, the UK has one of the lowest numbers of doctors per capita in Europe (EU average of 4.2).^2^, ^3^, ^4^ Combined with an unprecedented backlog of care exacerbated by the COVID-19 pandemic, this has resulted in an exhausted and burnt-out workforce. The 2023 General Medical Council (GMC) survey reported that 21% of doctors were at risk of burnout, with 66% of doctors reportedly working beyond their rostered hours, one in three feeling unable to cope with their workload, and doctors citing workload pressures or inadequate staffing as the most common cause of compromised patient safety.^5^

In June 2023, NHS England published the NHS Long Term Workforce Plan (LTWP), pledging to double the number of medical student training places by 2031.^6^ This is the first comprehensive policy designed to plan strategically for the future of the NHS workforce in England. The plan also committed to novel schemes to train medical students, such as 4-year (accelerated) undergraduate medical degrees (AUMDs) and the medical doctor degree apprenticeship (MDDA). MDDAs have since been paused and, despite work to develop an AUMD curriculum set to commence in 2026, little progress has yet to be published.^7^^,^^8^

While the need for long-term planning and retention was acknowledged and welcomed by the medical profession, other aspects of the LTWP faced more robust challenge, especially regarding an apparent lack of detail for implementation.^9^, ^10^, ^11^ Following a ‘change NHS’ consultation, the new government stated its intention to refresh the LTWP in late 2025. This was to follow publication of the NHS 10-Year Plan, initially due to be published in spring 2025 but recently released in July 2025.^12^, ^13^

To date, there has been no analysis of the challenges to expanding medical student placements, as outlined in the LTWP, and questions over feasibility remain. This prompted a scoping review asking, ‘What are the challenges of expanding the number of medical students in England as part of the NHS LTWP?’ and searched existing literature, summarising challenges and identifying gaps for further research, with the aim of guiding future research and policy.

## Methods

It was anticipated that the search would include a range of different types of literature; therefore, a scoping review was felt to be the most appropriate methodology, allowing literature to be included regardless of its type or quality, facilitating a wide, malleable and reproducible approach.^14^ This review follows methodology set out by Arksey and O’Malley, guidance from the Joanna Briggs Institute (JBI) for scoping reviews, and conducted in accordance with an *a priori* protocol which can be found in appendix A.^15^^,^^16^

### Identifying the research question

The final research question agreed upon was: ‘What are the challenges of expanding the number of medical students in England as outlined in the NHS LTWP?’

### Identifying relevant studies

Seven databases were searched to find relevant articles, chosen to focus on medicine, social sciences and health policy. MEDLINE, Web of Science, Scopus, ASSIA, TRIP database, Social Science Database and EBSCOhost were searched, using the search strategy outlined in Table 1. All databases were searched on 4 November 2024, and then again on 7 January 2025, ensuring that recently published articles were included, except for the TRIP database, which was only searched on 9 November 2024.

**Table 1:** MEDLINE search strategy.

	Search terms
1	(NHS Workforce Plan or NHS Long Term Workforce Plan or LTWP) and (doctor or medical student or physicians)
2	((increas* or expand* or doubl*) adj5 (‘medical school’ or ‘medical schools’ or ‘medical student’ or ‘medical students’))
3	((‘medical’ or ‘doctor’) adj5 apprentice*) or ‘MDDA’
4	((‘4 year’ or four year or accelerated or fast track) and ‘undergraduate’ and (medical degree or medicine degree or doctor))
5	(1 OR 2 OR 3 OR 4) AND NHS

Searches were then undertaken to find relevant grey literature. Websites of stakeholder organisations likely to report on medical student expansion were searched using their own search tools, or when not possible, by a Google site search. Organisations searched included the British Medical Association (BMA), UK Foundation Programme Office (UKFPO), the Medical Schools Council (MSC), NHS Confederation, the Health Foundation, General Medical Council (GMC), Royal College of Physicians (RCP), Royal College of Physicians of Edinburgh and the Academy of Medical Royal Colleges.

### Study selection

Articles were downloaded and duplicates removed. Studies were subjected to a three-stage screening process. Both ES and RhC independently reviewed each source title against the inclusion and exclusion criteria outlined in the protocol. Any disagreements were discussed between the reviewers, before being repeated with abstracts and then subsequently full-paper screening.

Inclusion criteria included articles written in English, and those referencing medical schools training students to work as doctors in the NHS in England. Undergraduate medical education changed dramatically in 2020, as a result of the COVID-19 pandemic, changing many final-year clinical placements and graduation requirements. Therefore, articles published prior to 1 January 2021 were excluded, avoiding legacy papers related to this unique pandemic period being reviewed.

Although expanding medical student numbers may inevitably worsen specialty postgraduate training bottlenecks, this issue was deemed too critical and extensive to be included and was reserved for a separate review. Therefore, references pertaining to specialty or postgraduate training were excluded. Graduate-entry medical courses were included. Changes to medical school curricula not relating to increasing the number of students or shortening/changing the degree length were excluded.

To gain a complete picture of the challenges raised, published literature was included regardless of type or quality, except for short comment pieces, such as letters or rapid responses, or news articles derived from a single press release. News articles providing multiple stakeholder perspectives relevant to the research question were included.

**Fig. 1:**
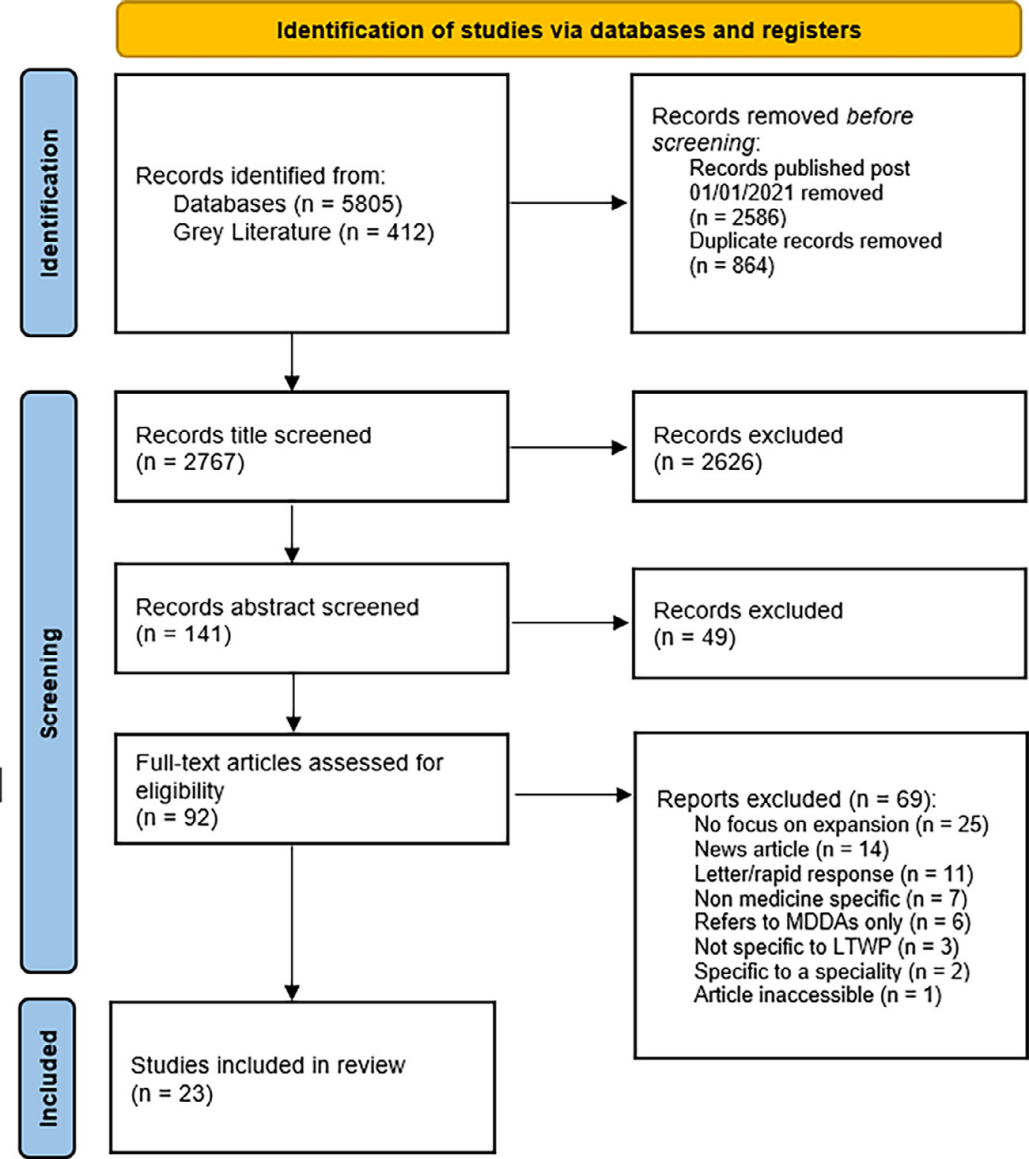
PRISMA diagram for the scoping review.

Following the news that government was pausing the MDDA programme, it was agreed to change the exclusion criteria to exclude articles solely relating to MDDAs. At the time of writing, there is no confirmation as to the fate of AUMDs, which were therefore still included in the review. A PRISMA flow diagram displaying how articles were screened is shown in Fig. 1.

### Charting the data

Each article was charted into an extraction tool as guided by Arksey and O’Malley.^15^ Articles were additionally categorised in reference to the expansion of medical students, AUMDs, or both. In addition, conclusions and challenges identified by each paper were noted. Appendix B contains the final extraction of all 23 papers.

### Collating, summarising and reporting the results

A set of 12 repeated challenges were identified, and papers were subsequently tagged according to the challenge(s) raised and then grouped together into themes, allowing for further analysis.

## Results

**Fig. 2:**
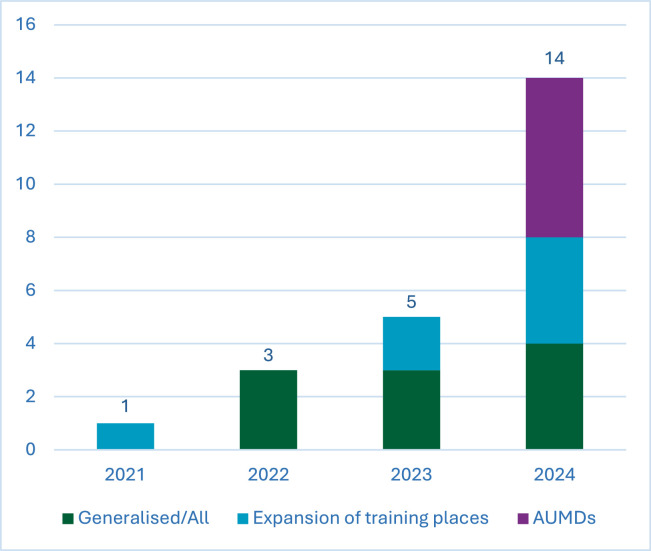
Date of publication of included articles, categorised by topic discussed.

23 articles were included in the scoping review. Increasing numbers of articles were published with each progressive year searched, with over half (61%) published in 2024, as shown in Fig. 2. Six papers focused solely on AUMDs, seven on generalised expansion and ten papers focused on both.

**Fig. 3:**
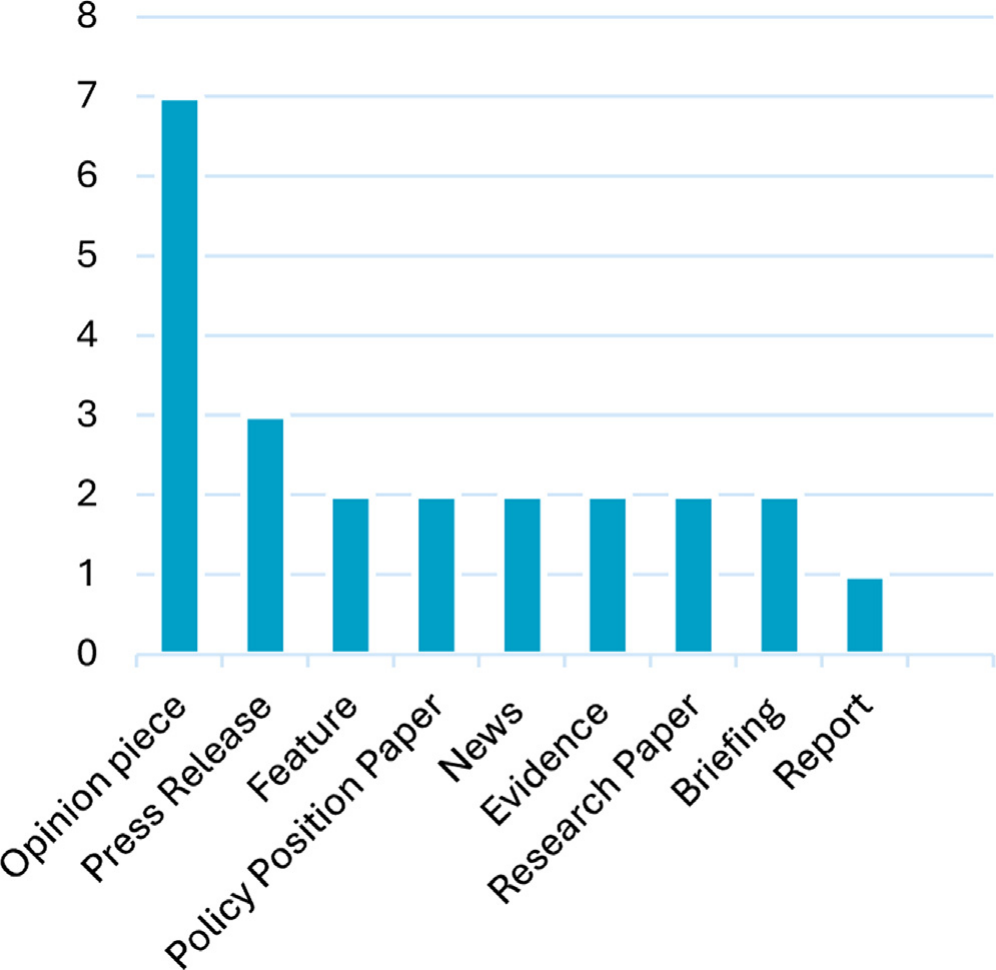
Breakdown of literature by type of articles included.

Only two articles were peer-reviewed research papers: a retrospective case control study, with a sample size of *n*=1,769, and an ‘invited review’, sample size *n*=60.^10^^,^^17^ There were seven opinion pieces, making up the largest proportion of articles. Opinion pieces included commentaries, editorials and analysis articles. Three press releases were included. Only two news articles were considered to have provided enough perspectives to have been included in the review.^9^^,^^18^ The full breakdown of literature type included is found in Fig. 3.

Funding was the most frequently raised challenge, mentioned in 54% of all papers, followed by a lack of clinical academics and insufficient stakeholder engagement, mentioned in 39% and 35% of papers respectively. Paucity of clinical placements and risks to Widening Access to medicine (WA) were both identified in 30% of literature. A single policy position paper did not directly identify any challenges, as it was written before LTWP publication.^19^

The frequency each challenge was raised is found in Table 2, grouped by theme.

**Table 2:** Challenges raised by articles in the review.

Themes	Challenges	Inclusion in articles *n*, (%)
Funding	Funding lacking/Unclear	12 (52)
Capacity	Educator/Academic shortage	9 (39)
	Placement capacity concerns	7 (30)
Political	Lack of stakeholder engagement	8 (34)
	Short-termism/Political concerns	5 (22)
Educational	Risks to widening access/Participation	7 (30)
	Limited degree transferability/Risk of two tier doctors	6 (26)
	Risk to patient safety	6 (26)
	Student burnout/Welfare risk	4 (17)
Practical	Implementational uncertainty/Lack of detail	4 (17)
	Unrealistic expectations/Criticisms ignored	3 (13)
	Educational vs. service provision prioritisation	2 (9)

### Funding challenges

Insufficient and/or opaque funding was a concern raised in 12 articles. Macdougal *et al*^20^ highlight how investment in medical education has faltered, making up only 2.8% of NHSE’s budget, compared to 5% in 2006/7. Anxiety around allocated funding being insufficient to cover all costs of expansion was frequently noted,^18^^,^^21^, ^22^, ^23^, ^24^ with concerns of increased salary costs of a larger workforce entering postgraduate training putting additional fiscal pressure on the NHS budget.^21^^,^^25^^,^^26^

Questions remain as to whether the current model of tariff funding of undergraduate medical education, with funding given directly to placement providers, works effectively. Issues surrounding transparency and accountability suggest more aggressive policing of tariff spend could free up additional revenue, reducing barriers to expansion from the treasury.^27^

### Capacity challenges

Serious concerns were voiced in the literature surrounding insufficient physical placements for medical schools to expand into which was identified as a key limitation in NHSE’s modelling.^10^^,^^21^^,^^23^^,^^28^, ^29^, ^30^ The Department of Health and Social Care (DHSC) plans for new medical schools to be built in historically under-doctored areas; however, these tend to be areas that may struggle with limited infrastructure to train more students easily using conventional training models.^23^^,^^27^

Training more medical students requires more clinical academics, teaching fellows and dedicated teaching responsibilities in doctors’ job plans. A global clinical academic shortage was frequently highlighted in the literature.^10^^,^^11^^,^^28^^,^^29^^,^^31^^,^^32^ Waldock *et al*^17^ in their retrospective cohort study using GMC National Trainee Survey Data, estimate that only approximately half of doctors undertaking clinical and academic training during 2018–2021 ended up as clinical academics in medical schools. The MSC reports that there has been a 25% reduction in senior clinical lecturers in the last decade and a 4% decline in academics more generally, despite growth in student numbers.^23^ Macdougall *et al*^20^ criticise stakeholders who, despite acknowledging the problem, fail to follow up with action, beyond broad statements such as ‘capacity is needed’. The frequent restructuring of organisations with strategic responsibility for training is frequently blamed for why action to tackle this has been slow.^20^

### Political challenges

Workforce planning needs time and certainty, and literature expressed frustration that the LTWP lacks an implementation strategy beyond initial steps and is too focused on the short term.^20^, ^21^, ^22^^,^ ^26^

There was a large amount of disenfranchisement in the literature, particularly by stakeholders, regarding the lack of consultation in the plan, especially surrounding the introduction of AUMDs.^20^^,^ 33, 34, 35

### Educational challenges

There was widespread concern that AUMDs may create ‘two tiers’ of doctors and medical students. By falling short of the 5,550 hours required to meet European standards, more rapidly trained UK medical students may end up with qualifications that are not accepted abroad, consequently trapping graduates in Britain.^9^^,^^27^^,^^29^^,^^36^^,^^37^

Potential safety concerns exist for AUMDs, with many arguing that reducing medical training by 20% may have a negative impact on patients.^31^^,^^33^, ^34^, ^35^^,^^38^ Palmer and Rosen are also worried that a reduction in entry requirements as medicine becomes less competitive, would lower the (perceived) quality of UK doctors.^24^

There are additional fears that AUMDs will not widen participation as suggested. Although AUMD students will graduate with less debt, there would be little ability for paid work during a compressed degree, making an AUMD course paradoxically less affordable and potentially entrenching WP students in inequality via debt.^9^^,^^10^^,^^33^^,^^37^

Medical students are at a higher risk of stress, burnout and depression than their non-medical student counterparts, and there was significant anxiety that a highly intensive AUMD course could compound this.^10^^,^^33^ Lim *et al.*^37^ highlight evidence from America, where 25% of students on accelerated MD programmes voluntarily extend their education due to high levels of stress.

### Practical challenges

The literature challenges the LTWP as being overly ambitious, with the National Audit Office noting the target to ‘double’ numbers being at the upper limit of what is theoretically possible.^25^ NHSE has also been accused of failing to realise the ‘multiple layers of governance’ involving multiple organisations slowing down delivery.^26^

There remains a large amount of uncertainty in the literature, around plan implementation, especially when discussing how AUMDs will work in practice.^11^^,^^22^^,^^37^ Palmer and Rosen highlight the ‘consequences of ramping up training are largely untested’ and even to key stakeholders, details remain ambiguous.^10^^,^^24^

## Discussion

### Refreshing the LTWP

The DHSC will need to consider challenges raised in literature when reviewing the LTWP later this year. It also remains uncertain whether the current government will maintain its commitment to doubling the number of medical students over the timescales initially stated. The future of AUMDs and any implementation strategy remains unclear, as does the permanence of the current pause of MDDA courses.

A common theme running through the literature is the lack of influence of stakeholders and medical professionals in workforce planning. NHSE and DHSC should act upon the concerns of staff, students and universities accordingly. As suggested by Macdougal *et al.,*^20^ a national strategy to support clinical academics and address capacity of clinical placements is urgently needed, in close alignment with the expansion of postgraduate training.

Gateway year courses, adding an additional year to the start of medical school, are proven to make medicine more accessible, so it is unclear how the opposite strategy of reducing courses by a year will be successful in widening access.^39^^,^^40^ Other suggestions to support WA include less-than-full-time study, while extending the course over a longer time period, and an urgent review of medical student funding to tackle the ‘unliveable’ NHS bursary.^27^^,^^41^

Setting up an AUMD course does not train significantly more doctors than a traditional course, save from its first graduating cohort. The main advantage of reducing course length by a year would be in reducing the cost per doctor trained. Starting work earlier could have unintended consequences, leading to earlier burnout or retirement, which may result in little significant net gain in numbers. The LTWP makes little mention of encouraging more graduate-entry programmes, which are frequently more oversubscribed than undergraduate entry courses; some graduate-entry courses have even been shut down in recent years.^42^

### Areas for future research

This review did not identify any literature reviewing the impact on learning outcomes of expanding student numbers, particularly in an overstretched and overburdened NHS, pressured for increased service provision.

Limited literature was identified on how medical schools plan to manage any expansion. Previous identification of additional placement capacity could now be outdated, for example, the last geographical snapshot of undergraduate GP teaching was published in 2014.^43^

Eventually, a saturation point for the current model of traditional placement capacity will be reached, and alternative models, such as longitudinal integrated clerkships, and a greater use of technology and high-fidelity simulation will be necessary if further expansion is to go ahead.^44^^,^^45^ These options were trialled during the start of the COVID-19 pandemic, with some success, but concerns remain regarding learning outcomes were these to replace, rather than complement, conventional clinical teaching practice.^46^, ^47^, ^48^

Certainly, further consideration of AUMDs is required before implementation to ensure that appropriate learning outcomes are covered, so NHSE does not risk training under-prepared practitioners or, worse still, compromise patient safety. Limited real-world data will be available to reassure the public in this regard until initial pilot programmes graduate students. However, some preliminary studies should precede implantation, such as surveying current graduate-entry students on how they feel they would have coped with a 4-year course as a school-leaver.

### Limitations of the review

This was not a systematic review and although the search strategy was broad by design and necessity, it was undeniably less robust, with the exclusion criteria potentially removing some relevant articles. One of the main limitations of a scoping review is there is no assessment of the quality of sources, nor assessment of bias. Political sensitivities around this topic tend to encourage rhetoric, with many simply representing personal opinion. As mentioned previously, only two sources were peer-reviewed research. Given the significance of the plan to the future of the NHS and medical education, the limited number of studies considering various aspects of the plan is somewhat surprising. It is possible that this could be attributable to this being the first such plan of this nature, certainly in terms of far-reaching scope and magnitude, or perhaps that the intersection between medical education and health policy is somewhat underrepresented within academic research.

Politics and policy can also change very quickly, the recent announcement from government to abolish NHS England being a recent case in point. It is still unclear how this will impact undergraduate medical training and workforce planning at the time of writing.^49^ This review focuses on the LTWP, which is policy for England only. As healthcare policy is devolved, it may not be directly applicable across the other three nations of the UK.

The exclusion criteria purposefully removed articles relating to postgraduate medical training, such as the specialty bottleneck, but considerations for postgraduate training is critical in updating any LTWP. The recently published NHS 10 Year Plan outlines a planned expansion of 1,000 new specialty training posts over the next 3 years; however, given the scale of the bottleneck, this is unlikely to resolve this national shortage fully.^13^ Perhaps this will be tackled in NHSE’s recently announced postgraduate training review, which seeks to address concerns and improve training pathways for UK doctors.^50^

## Conclusions

In common with many policy proposals, obstacles will inevitably appear to restrict implementation as initially planned, and the LTWP is no exception. However, many valid concerns have been raised by the medical profession and other stakeholders. NHSE and DHSC should be encouraged to listen carefully to these. There is a desperate need to provide further clarification around strategy and funding if a new LTWP is to be successful in fixing the workforce crisis more broadly.

The medical profession also has a responsibility to continue to highlight workforce challenges and raise concerns, but also to better understand its own workforce; investigate how to train more students than ever before, while maintaining quality of experience and widening access, ensuring the profession better represents the population it serves. It must continue to train medical students to a high standard, equipping them for future clinical practice while simultaneously providing adequate wellbeing support throughout their careers.

As discussed, many issues raised are largely opinions, with limited evidence to better inform decision-makers. As the government commits to revitalising the LTWP, but with little detail in the public domain regarding what aspects of this might change, it is vitally important that the medical profession remains engaged and proactive in providing evidence where possible and in carefully and critically reviewing the revised plan when published. The exact timeline for the publication of the new LTWP remains unclear; however, it is now expected in late 2025. Regardless of timings, research focusing on the intersection between healthcare, workforce planning and medical education is needed, to provide robust evidence to guide future plans and decision making.

## CRediT authorship contribution statement

**Eli C. Sassoon:** Writing – original draft, Methodology, Formal analysis, Conceptualization. **Rhiannon C. Craig:** Validation, Writing – review & editing. **David G. O’Brien:** Writing – review & editing, Supervision.

## Declaration of competing interest

The authors declare the following financial interests/personal relationships which may be considered as potential competing interests:

Eli Sassoon reports a relationship with British Medical Association that includes: board membership and travel reimbursement. ES and RhC are both medical students at the University of Nottingham. DOB is a professor of medical education and was involved in the setting up of a new medical school (Lincoln Medical School). ES is a member of BMA Council and immediate past member of the BMA’s Medical Student Committee Executive.

If there are other authors, they declare that they have no known competing financial interests or personal relationships that could have appeared to influence the work reported in this paper.

## Appendix Supplementary materials

Image, application 1
