# Supplementary material for: The challenges of expanding medical student numbers in the UK: A scoping review
Source: Future Healthc J. 2025 Jun 28;12(3):100278. doi: 10.1016/j.fhj.2025.100278 (PMC12318329; doi:10.1016/j.fhj.2025.100278)
Supplement: Supplementary file 1 [file mmc1.docx]

# APPENDICES

## Appendix A: Priori Protocol

The challenges of expanding the number of medical students as part of the NHS Long Term Workforce Plan – a scoping review protocol.

### Abstract

**Objective:** The objective of this scoping review is to identify the challenges around the proposed expansion of the NHS Long Term Workforce plan (LTWP) and map what research has been undertaken on this topic.

**Introduction:** The NHS in a staffing crisis, with not enough doctors to sustain the UK’s ageing population. The NHS published its LTWP with the intention to improve this, which includes commitments to expanding the number of medical students and the introduction of new and more controversial ways of training doctors.

**Inclusion criteria:** This review will consider literature relating to the expansion of UK medical schools as detailed in the NHS LTWP. It will focus on the challenges identified, including practicality and any potential risks to patient safety.

**Methods:** Seven databases will be searched in addition to some grey literature, and articles will not be excluded based on their type or quality. Only papers published in English after 1^st^ January 2021 will be included. The titles and then abstracts will be screened. This will be followed by a full-text screening, with light touch assistance of a second reviewer, followed by data extraction.

### Introduction

The NHS faces a workforce crisis, with a 6.9% vacancy rate for medical staff in England, equal to 10,745 Full Time Equivalent (FTE) [1]. When compared to other countries the UK lags behind with one of the fewest doctors per person in the OECD, with 3.2 doctors per 1,000 people. For comparison, Ireland, sits at 4 doctors per 1,000 [2]. These staffing shortages combined with a backlog of care has resulted in an exhausted and burnout workforce with the latest GMC survey reported that 21% of doctors were at risk of burnout [3].

This is partially as a result of the UK’s ageing and expanding population. The UK life expectancy has increased by 13 years since the NHS was first founded in 1948 and the UK population has grown dramatically [4].

On the 30^th^ of June 2023, NHS England published the ‘NHS Long Term Workforce Plan’ (LTWP) This was the first time the government had asked the NHS to put together a blueprint that strategically plans for its future staffing [5]. The plan is divided into three core priorities: Train, Retain, and Reform. The first component (on training) outlines plans on doubling the number of medical students by 2031/32 by introducing Medical Doctor Degree Apprenticeships (MDDAs), and by establishing four-year Accelerated Undergraduate Medical Degrees (AUMDs).

However, the LTWP has not been without its controversies and criticism, with many questioning the practicality, cost-effectiveness and the safety of an expansion in this manner [6, 7] . There is already some existing literature on the LTWP but it is unclear what has been well researched, and what concerns of the plan need additional reviewing. There have been no identified scoping reviews on this topic, therefore the intention is to complete this review to systematically map the available literature around the ‘’Train’ section of the NHS LTWP. Specifically focusing on the challenges that are faced in the plans objectives to double the number of medical students, including introducing MDDAs and AUMDs. This can then lay the groundwork for additional research into this topic, to help shape the implementation of the plan and future NHS workforce planning. The most appropriate method to undertake this piece of research is a scoping review.

### Review question

The primary question is as follows: What are the challenges of expanding the number of medical students as outlined in the NHS LTWP?

### Inclusion criteria

Any medical school or medical training provider in the UK that trains students to become medical doctors to work in the NHS in the UK. This also includes training programmes that are not ‘conventional’, primarily MDDAs or AUMDs. Outcomes measured will include challenges including practicality and the safety of such an expansion.

### Exclusion criteria

Articles were only included if they were published after the 1st January 2021, to avoid any legacy papers around staffing and training during the Covid-19 pandemic being included in the study. As some aspects of the plan, such as MDDAs, had already been discussed and announced in advance of the plan being published, the search includes papers before the LTWP was published.

Although the post-graduate speciality bottleneck is a critical issue that has been raised in literature, it will be excluded in evidence selection, as it is too big a topic to study in sufficient detail given current resourcing and time constraints. This review will focus purely on the challenges with medical student expansion and not already increasing the number of qualified doctors. Any reference to speciality or postgraduate training will be excluded, as will any references to changes to medical school curricular that do not relate to increasing the number of medical students, or shortening/changing the degree and time spent in either academic or clinical learning environments.

Table 1: A PCC framework for eligibility of studies

| **Criteria** | **Determinants** |
| --- | --- |
| P – Population | Any UK based medical school or NHS setting involved in training medical students |
| C – Concept | Challenges with the expansion in the number of medical students, including new courses |
| C – Context | The NHS Long Term Workforce Plan |
|  | Contemporary medical education training initiatives in the UK, specifically MDDAs and AUMDs |

#### Types of sources

Given how recently the NHS LTWP has been published and in order to gain a full picture of the potential challenges that the plan may face, all published literature is to be included, with research not being excluded based on their type or quality. However: short comment pieces/letters/rapid responses will be excluded, as will any literature that is simply reporting on the publication of the LTWP or a news article that is published as a result of a single press release being put out by a single stakeholder organisation such as NHS England or the BMA. Any news articles that provides nuanced and multiple stakeholder perspectives relevant to the research question will be included. A selection of grey literature will also be searched and included, such as press releases from relevant stakeholder organisations.

### Methods

The proposed scoping review will be conducted in accordance with both the methods set out by Arksey and O’Malley [8] and following the guidelines set out by the JBI methodology for scoping reviews [9]. It will also use the Preferred Reporting Items for Systematic Reviews and Meta-Analyses extension for Scoping Reviews (PRISMA-ScR) checklist [10].

## Search strategy

This review undertakes a three-step procedure to search and identify published and unpublished studies on training. In the first instance, a limited search of MEDLINE and Web of Science was undertaken to identify and highlight some key articles on the topic. This guided the second search, with the text contained in the titles and abstracts of the first, used to develop a draft search strategy. This draft search was tested once again on MEDLINE and Web of Science. Following this, the search strategy was then refined, to ensure it contained a wide enough scope, and it will now be used in all databases to find literature ready for screening. All searches will be completed in line with my institutions policies on literature reviews and advice received from my institution librarian.

Seven databases were chosen to identify relevant literature, and these were chosen to focus on medicine, social sciences and health policy. These databases are: MEDLINE, Web of Science, Scopus, ASSIA, TRIP database, Social Science Database and EBSCOhost. As an example of the search strategy, a complete search that will be used to search the MEDLINE database can be found in Table 2: MEDLINE search strategy. For any database that does not allow the use of the “adj” or “near” search operators, these search operators will be replaced by the Boolean variable “and”.

Table 2: MEDLINE search strategy

|  | **Search terms** |
| --- | --- |
| 1 | (NHS Workforce Plan or NHS Long Term Workforce Plan or LTWP) and (doctor or medical student or physicians) |
| 2 | ((increas* or expand* or doubl*) adj5 ("medical school" or "medical schools" or "medical student" or "medical students")) |
| 3 | (("medical" or "doctor") adj5 apprentice*) or “MDDA” |
| 4 | (("4 year" or four year or accelerated or fast track) and "undergraduate" and (medical degree or medicine degree or doctor)) |
| 5 | (1 OR 2 OR 3 OR 4) AND NHS |

Grey literature will also be included. This will involve searches of the websites of reputable organisations that are likely to have published data, reports or policy positions on the LTWP and more specifically, on the expansion of medical student numbers. Examples of these are likely to include the royal college of Physicians, the Medical Schools Council, the BMA, NHS Confederation and the UKFPO.

## Study/Source of evidence selection

Following the search, all literature citations will be uploaded to Endnote 21.4. Duplicates will then be removed. The titles of all the papers will then be screened any irrelevant studies removed, this will be guided by the PCC framework outlined in Table 1: A PCC framework for eligibility of studies. Following this, the abstracts of all remaining sources will be screened and once again compared against the inclusion criteria for the review. If a source does not have an abstract for example, a press release it will automatically pass through abstract screening. Potentially relevant sources remaining at this point will be retrieved in full and assessed in detail against the inclusion and exclusion criteria. Each of these three stages will be screened independently by two separate reviewers, who after each stage will meet and discuss the papers. Any disagreements at this point will be considered at length and if the two reviewers cannot agree, the paper will be referred to a third reviewer for a final decision. The results of the search, and the study inclusion process will be reported in full in the final scoping review and presented in a PRISMA flow diagram [10].

## Data extraction and charting

Data extraction and verification will be undertaken by one reviewer, although unideal, this due to time constraints. The data will be extracted from papers and extracted in a tool to record key information about the source, including key findings relevant to the research question, and information about the type of source and methodology. A draft of the headings in the data extraction tool can be found in Table 3: Proposed data extraction form, which will be stored in Microsoft Excel. Any further refinement and updates to the form will be highlighted in the final dissertation.

Table 3: Proposed data extraction form

| **Title** |  |
| --- | --- |
| Author(s) |  |
| Year published |  |
| Publication location OR institution publishing |  |
| Type of article | *e.g. Research paper, policy report, short communication* |
| Methodology | *e.g. Quantitative, Qualitative, opinion piece* |
| Empirical data used?  (If yes: primary/secondary data? and N=) |  |
| Inclusion of which UK nations? |  |
| Study population |  |
| Study method and design |  |
| Specific mention of the LTWP? |  |
| Focus(es) from the ‘train’ section | *e.g. MDDAs, AUDs, doubling number of places,* |
| Conclusions |  |
| Challenges identified | *Challenges that the paper identifies with any part of the LTWP* |
| Comments |  |

## Data analysis and presentation

By definition, scoping reviews do not synthesise the results of any included sources but rather attempts to chart out what research has been completed on a topic. This is especially useful with fast moving pieces of research where additional literature is regularly being published. There is unlikely to be much, if any, quantitative data discovered in this mapping and so in the initial stage of data analysis the information will be coded into categories relating to source type, which parts of the UK it is relevant to and the challenges that have been identified. Although a narrative report will be written to summarise the challenges raised in the literature in a thematic way, this will not constitute a thematic synthesis as this would be far beyond the jurisdiction of a scoping review and would requires a systematic review of any qualitative evidence.

### Author contributions

Eli Sassoon wrote the original manuscript. With thanks to Dr Stephen Agius and Professor David O’Brien for providing advice and guidance with regards to scoping review methodology.

### Declarations and Conflicts of interest

Eli Sassoon is a medical student at the University of Nottingham (2022-2027) and a member of the BMA UK Council (2024-2026) and is an immediate past executive member of the BMA’s Medical Students Committee.

### References

1. Workforce and Estates Team: **NHS Vacancy Statistics England, April 2015 - March 2024, Experimental Statistics**. In*.* Edited by NHS England: NHS Digital; 2024.

2. OECD: **Doctors (overall number)**; 2023.

3. General Medicine Council (GMC): **The state of medical education and practice in the UK. Workplace experiences 2024**. In*.* London; 2024.

4. Office for National Statistics (ONS): **National life tables – life expectancy in the UK: 2020 to 2022**. In*.*; 2024.

5. NHS England: **NHS Long Term Workforce Plan**. In*.* London; 2023.

6. Ferreira T, Collins AM: **The NHS Long Term Workforce Plan: an ambitious leap or a misstep?** *Journal of the Royal Society of Medicine* 2023, **116**(11):368-370.

7. Geary U, McKee M, Petty-Saphon K: **Mind the implementation gap: a systems analysis of the NHS Long Term Workforce Plan to increase the number of doctors trained in the UK raises many questions**. *British Medical Bulletin* 2024, **150**(1):1-10.

8. Arksey H, O'Malley L: **Scoping studies: towards a methodological framework**. *International Journal of Social Research Methodology* 2005, **8**(1):19-32.

9. Micah DJ Peters CG, Patricia McInerney, Zachary Munn, Andrea C. Tricco, Hanan Khalil: **Scoping Reviews**. In: *JBI Manual for Evidence Synthesis.* 2024.

10. Tricco AC, Lillie E, Zarin W, O'Brien KK, Colquhoun H, Levac D, Moher D, Peters MDJ, Horsley T, Weeks L *et al*: **PRISMA Extension for Scoping Reviews (PRISMA-ScR): Checklist and Explanation**. *Ann Intern Med* 2018, **169**(7):467-473.

## Appendix B: Data Extraction Spreadsheets

| **Paper** | **Author(s)** | **Year published** | **Publication location or institution publishing** | **Type of article** | **Methodology** | **Empirical data used?**  (if yes - primary/secondary data? and N=) | **Study population** | **Study method and design** | **Inclusion of which UK nations?** | **Specific mention of the LTWP?** | **Focus(es) from the ‘train’ section** | **Conclusions** | **Challenges identified** | **Challenges identified (**category) |
| --- | --- | --- | --- | --- | --- | --- | --- | --- | --- | --- | --- | --- | --- | --- |
| BMA Briefing - Medical Students | BMA Medical Students | 2023 | BMA (British Medical Association) | Briefing | N/A | N/A | N/A | N/A | UK | Published before LTWP | Expansion of training places | - Calls for doubling number of medical school places - But this will not be possible without expansion of the consultant and medical educator workforce, of existing university capacity and infrastructure, and of hospital and teaching facilities, as well as the construction of new medical schools - Ensure that increases in medical student places see an increase in WP proportion places - Medical students are at increased risk of stress, burnout and depression, | The need to expand the  - consultant and medical educator workforce  - existing university capacity and infrastructure, hospital and teaching facilities - constructions of new medical school There are other issues facing medical students such as:  - Medical student finance troubles (NHS bursary) - 19% of medical students knew how to access welfare support on placement | Educator/Academic Shortage, Placement Capacity Concerns, Risk to Widening Access, Student Burnout/Welfare Risk |
| Doctors vote for BMA to oppose four year medical degrees and apprenticeships | Gareth Lacobucci | 2024 | BMJ (British Medical Journal) | News | N/A | N/A | N/A | N/A | UK | No | MDDAs + AUMDs | -BMA representatives voted against MDDAs and AUMDs proposals at their annual representative meeting (ARM) - A student argued that the government should be funding WP schemes within medical schools, giving “proper” NHS bursaries, and providing funding for international medical students. - One Medical Academic argued that diluting medicine is bad, but that he didn't completely support the motion to completely oppose all MDDAs and AUMDs. The degrees must, however, ensure that all new proposals are evidence based, evaluated and retain international recognition. | - Concerns that these degrees will not meet the required hours needed to be recognised abroad, trapping students here in the UK.  - Concerns about the university accredited 5500-hour requirement for a primary medical degree. | Risk to Widening Access, Limited Degree Transferability / Risk of Two Tier Doctors |
| RCP responds to NHS England proposal for four-year undergraduate medical degrees in England | Royal College of Physicians | 2024 | Royal College of Physicians | Press Release | N/A | N/A | N/A | N/A | England | Yes | AUMDs | - When AUMDs were announced as part of the LTWP RCP said they wanted to scrutinise any proposals to ensure standards were upheld and were practical.  - College urged NHSE to release details on how five years will fit into four so same number of taught and learning hours are achieved as in a five-year course. - RCP says there cannot be a two-tier doctor’s system and much meet standards set out by the GMC and Royal Colleges. | - no information on engagement with stakeholders or consultation with the RCP occurred with the announcement of AUMDs - Negative impacts of trying to fit 5 years into 4 are: reduced knowledge and skill acquisition and retention and effects on mental health and wellbeing.  - Inhibit NHS England ability to widen access | Lack of Stakeholder Engagement, Risk to patient safety, Risk to Widening Access, Limited Degree Transferability / Risk of Two Tier Doctors, Student Burnout/Welfare Risk, Funding Lacking/Unclear |
| Four year medical degrees in the UK | Gabrielle M Finn, Megan E L Brown, Paul A Tiffin | 2024 | BMJ (British Medical Journal) | Opinion piece/Commentary/Editorial | N/A | N/A | N/A | N/A | England | Yes | AUMDs | - Existing curricular will have curricular bulges where stuff has been added, but little has been removed. These could be trimmed to focus on knowledge and skills needed for a digitalised health system.  - Also scope to reduce the holidays during preclinical years (18-20 weeks currently), but this would time for paid work | - Absence of evidence it will be harmful is not equal to absence, and reducing medical training by 20% will have unintended but predictable consequences.  - Risks underdevelopment of critical skills including: communication, empathy and other such soft skills may link to issues with patient outcomes. | Risk to patient safety, Short-termism/Political Concerns |
| Is there a sufficient supply of clinical academics for UK medical schools? A retrospective cohort study | William J Waldock, Elizabeth Hughes, Jane Dacre, Amir H Sam | 2024 | BMJ Open | Research Paper | Quantitative (Retrospective Case Control study) | Yes.  Secondary data N = 1769 | Registered UK doctors with academic training and have CCTed | - GMC statisticians used the GMC annual national trainee survey to identify potential supply of doctors from 2012-22 who were academic trainees and CCTed. | UK | Yes | Expansion of training places | - Half of fully qualified academic doctors do not progress into clinical academic work in UK medical schools and not staying in clinical academia  - Unclear if it is because there are no jobs, or they don’t want the jobs on offer - There is a significant discrepancy between the number of clinical academics per 1000 students between medical schools - which could widen the gap between research carried out by various uni's, career development, and widen health inequalities,  - Further study into gather more comprehensive data on clinical academics, study into the variation into academic trainees by medical school. - Better incentive frameworks for clinical academics need to be introduced through remuneration, accommodation, or allowances to work remotely in more affordable locations. | - Push factors include burnout and pay similar to other doctors, and also because academic centres of excellence are normally expensive places to live and rase a family when living grant to grant.  - Pull factors include private industry having more interesting and better paid jobs, and more interesting and rewarding work in research policy outside of medical schools  - 25% reduction in senior clinical lecturer doctors over the last decade and a 4% decline in clinical academics more generally | Educator/Academic Shortage |
| Medical training at breaking point: will an increase in learners push the system over the edge? | Adele Waters | 2024 | BMJ (British Medical Journal) | Feature | N/A | N/A | N/A | N/A | UK | Yes | Expansion of training places | - One of the biggest challenges in the NHS is delivering medical education, doctors are passionate about it but they are finding it harder and harder. - NHS England are implementing an educator workforce strategy | - Insufficient capacity to train more doctors | Education vs. Service Provision Prioritisation |
| Education is essential for implementing the NHS workforce plan | Colin F Macdougall, Sarah Allsop, Christine Douglass, Lindsey Pope, Sophie Park, Robert K McKinley | 2024 | BMJ (British Medical Journal) | Analysis | N/A | N/A | N/A | N/A | UK | Yes | Expansion of training places | - Key to success of the LTWP is train more (retain and reform as well) but also reform education and training.  - Scale of ambition is unprecedented, and although these proposals apply to NHS in England expansion strategies have also been produced for Scotland and Wales. - Success at this scale requires a substantial expansion in capacity to train healthcare professionals - thus education, training, and workforce development must move up NHS priorities.  - Both LTWP and Educator Workforce Strategy (EWS) acknowledge more educators must be identified and recruited.  - Specific proposals have included minor initiatives such as the use of recently retired educators, expansions of specific teaching roles and consideration of ways to reduce costs of placement activities.  - Educator surveys have had specific recommendations are likely to echo what is already known and well established.  - To enact the LTWP policy makers must give education equal priority to service provision and research, with increased investment in med ed capacity and expertise in advance of a major expansion.  - Requirement for dedicated education expertise to lead, design, and evaluate implementation alongside integrated and resourced clinical services. Including education leadership  - Med ed experts could provide input into strategic policy making and analyse impact of educational investment.  - They propose a table of actions to strengthen medical education which includes: 1. include educators from diverse backgrounds in clinical & HE strategic decision making 2. provide funding/time/recognition to allow career educators to have time for training 3. providing protection time and funding ringfenced for educational activities 4. normalise education as central, resourced, timetabled and appraised as part of all clinicians’ work 5. invest in clinical educational research 6. take time to plan and deliver education rather than seeking short term solutions to current workforce crisis.  - Attitudes need to shift so that staff stop taking time "out" or "away" to train others. | - a year on from the landmark publications, progress has been slow especially on undergraduate expansion - only 555 have been allocated in the first 2 of the 8 planned years, so 6945 more are needed over subsequent 6 years.  - There is a current global crisis in medical education: UK, US, Australia all have chronic underinvestment in med ed - static numbers of clinical educators (and an ageing workforce), despite number of students increasing by 6% (and doctors in training by 20%) - This is affecting curriculum delivery - which is exacerbated by the fact that clinical educators in the NHS are under increased pressure to prioritise service provision over educational activities, resulting in bigger learner groups, reduced placement opportunities and fewer hands-on opportunities. - Key stakeholders (e.g. tMSC, BMJ commission on future of NHS, royal colleges) all recognise these challenges, but underestimate their scale and importance with broad statements such as 'capacity is needed'.  - Continual reconfiguration, reconstitution and dissolution of organisations with strategic responsibility for national healthcare education, training and workforce development has reduced the speed of policy change in this area (e.g. merger of HEE with NHSE) - England spends about £5bn on health education, but this is falling, from 5% of budge in 06/07, to 3% in 18/19, to 2.8% now.  - Decision making is short term, and as med ed is expensive it is not a priority in the current financial and political climate - education deemed less important that delivery of care leading to reduced budgets. They are deemed not as mutually dependent by competing for resources. - Staff who choose to take on key educational roles have unclear career pathways, inconsistencies in recognition and reward.  - Already questions if the ICSs and ISBs are ready or able to provide guidance on addressing local capacity of education - Don’t just focus on how to increase workforce numbers, work on how to increase and develop the educator workforce needed to train them.  The LTWP proposal for expansion of medical school places sets out several proposals that do not arise from educational evidence. The plans do not refer to educational journeys, relative cost effectiveness or long-term educational implications. | Educator/Academic Shortage, Education vs. Service Provision Prioritisation, Funding Lacking/Unclear, Placement Capacity Concerns, Lack of Stakeholder Engagement, Short-termism/Political Concerns |
| Scrap bursary for medical students in England, says Institute for Fiscal Studies | Éabha Lynn | 2023 | BMJ (British Medical Journal) | News | N/A | N/A | N/A | N/A | England | Not directly by name but mentioned. | Expansion of training places | - NHS bursary is a grant between 10-14k awarded to undergrad medical and dentistry students to cover tuition fees and maintenance costs replacing government student loans. (for postgrads this covers only partial tuition fees) - IFS thinks this funding would be better used in retention of already qualified doctors and replace NHS bursary with student loans, especially as medical students see funding drop during this time - those from the poorest households outside London would be eligible for £9,978 during their first 4 years, but only £6,248 in their final year when on the bursary.  - the Doctors Association UK have called for a liveable NHS bursary and says medical students should have access to the LSF | - Cost of NHS bursary will increase proportionally with expansion of the number of medical students | Funding Lacking/Unclear, Risk to Widening Access |
| Oral evidence: Workforce: recruitment, training and retention in health and social care, HC 893 | N/A | 2022 | Health and Social Care Committee | (Oral) Evidence | N/A | N/A | N/A | N/A | UK | Published before LTWP | Generalised/All | - 10,000 doctors short in the NHS so we should expand numbers of medical students - What is going wrong and blocking expansion? The tariff money paid by HEE to trusts to provide placements. 10x more expensive for a medical student than a nursing student (which it cannot cost more) it is a barrier to expansion from the treasury.  - The effect of burnout and poor morale in doctors is overflowing into medical students  - To increase doctors in certain areas you could build or expand medical schools in those areas (as medical students more likely to stay in that area) - A barrier to mature and graduate students training as a doctor is cost.  - Many medical students are earning while they learn, mostly in hospitality and if you made it quicker, they wouldn't be able to afford to do it because they need that time to work.  - Perhaps there is an option to do a part-time medicine course that takes longer. Which could widen access to medicine to those who couldn’t otherwise do it and improve social mobility.  - Money on funding medical students - is it going to travel reimbursement, placements, free accommodation etc - Generally we are doing training well, well respected degrees globally and shortening the curricula is a bad idea - public expect well trained doctors - that doesn’t mean curricula shouldn’t change as medicine evolves however.  - "On shortening the course, we get a lot of requests to put additional material into courses and different curricula. There are over 30 medical specialties and over 400 MLA conditions that we need to fit into our curricula. The undergraduate process is to create a potential doctor who can specialise thereafter, so the idea of shortening it is really quite difficult." - Prof Wilkes  - There may need to be expansions of GEC/GEM/JEP courses as many are on 5 year courses as they are particularly competitive.  - There may need to be a better focus on teaching medicine to more junior staff throughout medical school - Mental health of medical students is poor and needs to be tackled. | - You can shorten medical training and expect medical undergraduates to work faster and expect their trainers to teach them quicker and use prior competencies - but at the end of the day the same standard is expected, and you are cutting corners.  - Some of the severe doctor shortages are in areas where it is difficult to build a medical school  - NHS bursary needs to be looked at to see if it is supporting medical students in the right way.  - Impact on expansion on current medical student education of schemes and expansion - Clarification of how MDDAs will be formed | Risk to patient safety, Unrealistic Expectations / Criticisms Ignored, Risk to Widening Access, Implementation Uncertainty / Lack of Detail |
| The expansion of medical student numbers in the United Kingdom. | Medical Schools Council | 2021 | Medical Schools Council | Policy position paper | N/A | N/A | N/A | N/A | UK | Published before LTWP | Expansion of training places | - number of medical students should increase by 5,000 to 14,500 graduating a year, in the areas with capacity to provide high quality placements and in the regions where the shortage of doctors are most acute (although this is less than the total grads required because we can still recruit some from overseas) - There are 3-4 times as many applicants as medical school places and many well qualified candidates don't get a place.  - Medical schools are already developing ways to enhance clinical training that could support increase capacity such as using virtual learning opportunities  - Promotion and support of clinical academic careers will be essential as educators are needed to supporting learning - It is difficult to predict the exact number of doctors needed in the future, due to tech, expansion of MAPs, LTFW workers, etc.  - WP - innovative options such as the apprenticeship route and Less than full time education and conversion courses for healthcare workers can be developed.  - Medical schools are developing methos to allow more students on clinical placement that previously possible e..g through more specific timetabling and support from dedicated clinical teachers from a range of disciplines to optimise learning opportunities without affecting quality of educational experience. Also shift to use final year to prep students for practice, which makes students useful on the wards.  - 13 new medical schools with 250 new grads a year + expansions of existing med schools to an average of 200-250 grads a year would be required to hit their target of 5000. OfS thinks up to 17 med schools could expand. | - Cost - estimates of educating a UK medical student is £200k+, therefore an increase of 5,000 would be ~£1b (although there may be a way to reduce costs)  - Placement capacity - 'under-doctored' areas often have relative social depravation that may not have universities and NHS trusts in a position to hold a medical school. Although so medical schools are small and can expand, likely that the limiting factor is access to clinical placements especially in primary care - Small medical schools are less likely to be cost-effective (as lots of costs are fixed) and very large ones will struggle to expand to limitations on facilities and placements within a reasonable reach from the school - Clinical academics - responsible for course design, leadership and delivery of courses (in addition to research and clinical NHS services). Numbers have remained static over the last 10 years, and many are close to retiring. Also, significant issues in the academic pipeline in gender & ethnic diversity at specific progression points. More are needed | Educator/Academic Shortage, Placement Capacity Concerns, Funding Lacking/Unclear |
| RCP view on the NHS workforce: short- and medium term solutions | Royal College of Physicians, Royal College of Physicians of Edinburgh and Royal College of Physicians and Surgeons of Glasgow | 2022 | Royal College of Physicians | Policy position paper | N/A | N/A | N/A | N/A | UK | Published before LTWP | Generalised/All | - They were disappointed that the 2022 Health and Care Act did not include a legal duty for the gov to publish assessments of how many healthcare staff would be needed for demand. - There is a LTWP coming - just not yet.  - Lots of doctors are working LTFT and many consultants are close to retirement age.  - 3 ways to tackle workforce shortages: 1. increase number of staff 2. improve retention of those considering leaving 3. support approach to retirement to allow them to continue working appropriately.  - First step in the LTWP is to work out how many staff we need. (as best as possible) - It must include a range of short-, medium-, and long-term solutions to increase recruitment and retention.  - RCP has long called for the expansion of medical school places. They estimate doubling the number of medical students would cost £1.85bn (NHS currently spends 7.1bn on agency work) | None (written too early) | None (written to early) |
| Mind the implementation gap: a systems analysis of the NHS Long Term Workforce Plan to increase the number of doctors trained in the UK raises many questions | Una Geary, Martin McKee, Katie Petty-Saphon | 2024 | British Medical Bulletin | Research Paper | Qualitative (Invited review) | N/A | 60 individuals representing medical education and training stakeholders | - soft systems approach - ensuring that all sub-systems work together.  - On 18/09/23 tMSC brought together stakeholders to discuss how the proposed expansion of medical education set out in the LTWP can be implemented. - This included: NHS E & NHS WTE, NHS Employers, DHSC, GMC, BMA, UKFPO, UCAS, Nuffield Trust, Health Foundation and more - CATWOE mnemonic guided questions in groups of 6-8 people. (Customers, Actors, Transformation, Worldview, Owners, Environmental constraints) - Following the workshop a thematic analysis was carried out on the notes with identification of themes. | UK | Yes | Generalised/All | - Prospective evaluation of implementation is necessary - many gaps in LTWP - Doubling number of medical students has a major implication on human and capital resources, but number of clinical academics are falling progressively over time. - Need for clarity on what the plan will cost and where the funds are.  - There will need to be a big increase in primary care placement opportunities and GPs lack supervision capacity and facilities to take on new medical students - There were many drawbacks to the way in which expansion of medical students happened in 2016 especially around the resource intensive and inefficient bidding process. - needs to be collaborative going forward.  - process of policy developed in the UK has long been criticised for its failure to engage with those who have to implement policies.  Recommendations:  - Need for collaboration and coordinated planning between all partners involving medical schools to successfully implement the LTWP.  - Treasury and spending depts. fund and plan adequately for an expansion of medical school places - clarity on cost implications  - Establish additional postgraduate training places in a phased manner in line with medical student funding  - NHS England should publish proposals to create additional placement capacity, especially in primary care settings + investment in clinical educator staff and clinical facilities - NHS England needs to set out details MDDAs, how it will widen WP and details on how it will not threaten the reputation of med ed internationally. | - No approved MDDA scheme by the GMC and no clear determination who the benefits of the scheme are apart from the apprentices themselves. Major gaps identified: - Lack of subsequent postgraduate training places,  - A failure to consider the need for more educators (and facilities such as lecture theatres, labs [and there are difficulties in building anything]) - Lack of clinical placements [and questions if ICBs are able to turn clinical settings into clinical settings, especially relatively small populations] - Pitfalls with bidding process  - Uncertainty how MDDAs will work - these are revealing quite how unattractive the current funding and regulatory models are for universities. Fear that the scheme will not intrinsically widen WP, fear of inequity between students (including pay protection/debt/intercalation) - Potential negative impacts of AUMDS - risk not accepted abroad, can lead to financial pressures for disadvantaged students by reducing ability to work alongside their studies. Also risk of higher attrition and burnout. Could lead to narrowing of WP and increase failure & dropout.  - Is the medical school numbers realistic (1 in 6 enrolments into unis would be for health) | Educator/Academic Shortage,  Funding Lacking/Unclear,  Placement Capacity Concerns, Lack of Stakeholder Engagement, Risk to Widening Access,  Limited Degree Transferability / Risk of Two Tier Doctors,  Student Burnout/Welfare Risk |
| Shortening the medical degree | Helen Salisbury | 2024 | BMJ (British Medical Journal) | Opinion piece/Commentary/Editorial | N/A | N/A | N/A | N/A | England | Yes | AUMDs | - A consultation of the profession was widely expected so many commentators have been surprised by the announcement.  - Many students on undergraduate courses particularly WP students use holidays to work to keep afloat (as hard to have a part time job during medicine).  - Medicine hasn't become conceptually easier or less complicated in recent years - internet cannot replace grounding in science and critical thinking.  - feels as if it is about 'bumps on seats producing the next generation of doctors as quickly and cheaply as possible' - GEMs exist so it is possible to cover the curriculum in time and the GMC promises no lowering of standards. - cynic argued that this was done deliberately to not have the qualification recognised abroad so the recipient would have to remain in the UK. | - cheaper and quickly comes at the cost of less education, training, experience and expertise. Which can lead to poorer and less safe care for patients  - to be recognised in Europe primary medical qualifications require a minimum of 5500 hours of theoretical and practical training provided by or under the supervision of a university. (GEMs do this by sacrificing holidays). | Lack of Stakeholder Engagement, Risk to patient safety, Limited Degree Transferability / Risk of Two Tier Doctors |
| Statement on four-year medical degrees in England | Royal College of Physicians of Edinburgh | 2024 | Royal College of Physicians of Edinburgh | Press Release | N/A | N/A | N/A | N/A | England | No | AUMDs | - GMC have said on social media that AUMDs must meet existing standards if to be accepted - timing and method of announcement is against the spirit of engagement and consultation needed to lead change in UK medical education - NHS England is not the primary body for curricular design, especially when it deviates from well-established models of education and training of doctors (NHSE is responsible for delivery of healthcare) - If increasing student debt is the driver for AUMDs then NHSE should lobby the government to review financial support available for medical students over the duration of existing courses rather than try to reduce length of AUMDs in an unproven and controversial manner | - Lack of consultation for AUMDs  - Few details, but at face value the suggestion is concerning and a 4 year undergrad course would not go into sufficient depth for doctors. | Risk to patient safety, Lack of Stakeholder Engagement |
| NHS England's modelling for the Long Term Workforce Plan | Laura Cole, John Fellows, Hannah Kreczak, Natalie Low, Sumayyah Mian, Shivam Sood, Paul Wright-Anderson, | 2024 | National Audit Office (NAO) | Report | N/A | N/A | N/A | - NAO carried out an independent assessment of the modelling underpinning the LTWP.  - To consider whether NHSE constructed its models effectively and if they operated correctly in a technical sense to generate outcomes  - To consider if NHSE approach to workforce modelling and models are a reasonable bases for strategic workforce planning - No assessment of value of money for funding decisions  - Checked for errors and methodology. | England | Yes | Generalised/All | - Workforce modelling was complex and formed a pipeline. - The model with supply and demand projections for professions across 5 care settings and estimated a staffing shortfall  - The shortfall analysis done by the LTWP were good quality.  - Aspects of the modelling pipeline that NHSE designed are risky and modellers made many manual adjustments. This also included NHSE modellers moving data manually between the different components in the pipeline. These manual adjustments made risks model data and assumptions being inconsistent and increases risk of errors - NHSE documentation to the NAO varied in quality - and a lack of independent scrutiny and NHSE models showed a good understanding of the range of variables that will affect future size, but only NHSE only communicated a limited range of uncertainty in the published LTWP. - Some of the modelling assumptions may be optimistic given the change they imply. Some assumptions relate to historical trends which NHSE thinks will continue and others are more targets. | - The assumption on doubling the number of medical students is the top end of the maximum expansion NSHE thought was theoretically possible.  - The analysis did not include an assessment of the capacity constraints of expansion of training of this scale or the costs required to overcome any constraints. (NHSE worked to assess post LTWP publication) - Significant weakness which is the lack of integration between parts of the pipeline and manual adjustments. | Unrealistic Expectations / Critisisms Ignored, Funding Lacking/Unclear |
| The NHS Long Term Workforce Plan explained | Jessica Holden | 2023 | The King's Fund | Feature | N/A | N/A | N/A | N/A | England | Yes | Generalised/All | - Case for long-term change needed with an expected staff shortfall of 260-360k by 2036/37 - Plans for MDDA schemes | - Lots of detail but little connection to what the overall change will be for frontline staff in both education and healthcare.  - Funding required to implement the entire plan beyond 28/29 is unclear because gov. does not set out departmental budges beyond 3-5 years in advance.  - Consequences for day-to-day spending of the NHS as the salaries of new healthcare staff will cost (this is hard to predict especially with attrition etc)  - NHS England and Gov need to be realistic about the required capital investment in buildings and equipment too  - The increase in staff numbers will take to appear and will take time to be implemented into the system  - Risk that staff who are doing the training will not be well supported | Funding Lacking/Unclear, Short-termism/Political Concerns, Placement Capacity Concerns |
| The NHS Long Term Workforce Plan: what does it mean for BMA members? | British Medical Association | 2024 | BMA (British Medical Association) | Briefing | N/A | N/A | N/A | N/A | UK | Yes | Generalised/All | - Headline commitment of doubling the number of medical students is welcomed  - Imperative that MDDAs receive the same high standards of training than those in traditional pathways.  - If fully implemented the number of doctors per 1,000 would be around 4.3 which is equal to Italy and Sweden today (but below Germany and Austria) now - Step forward to even have a long term strategy and modelling (previously this didn’t exist) - Modelling is not broken down by speciality and there is a need for ongoing engagement with stakeholders such as royal colleges and the BMA. Expansion of public health and medical academic workforce is also needed. Gov have set out 2.4B to fund the 27% expansion for 2028/29 but no information as to how that funding will be allocated. - WP should not be focused on MDDAs, but should also be a priority for traditional training, Especially tackling financial and educational hurdles faced by school leavers and graduate entrances from underrepresented backgrounds. | - The plan lacks implementation strategy beyond the first steps - Arguably insufficient funding commitments to make the increase happen.  - Concerns regarding existing infrastructure e.g. teaching facilities and capacity of training.  - Lack of capital funding  - MDDAs and AUMDs are untested and there are significant concerns regarding their role in addressing the crisis  - Little scrutiny of the modelling and lots of the assessments done only looked at the approach of the modelling and not the scrutiny  - Unclear how you can shorten the already intensive medical training by a whole year. | Short-termism/Political Concerns, Funding Lacking/Unclear, implementational Uncertainty / Lack of Detail |
| The NHS Long Term Workforce Plan: an ambitious leap or a misstep? | Tomas Ferreira, Alexander M Collins | 2023 | Journal of the Royal Society of Medicine | Opinion piece/Commentary/Editorial | N/A | N/A | N/A | N/A | UK | Yes | Generalised/All | - Continual staff turnover disrupts care and dilutes collective expertise.  - UK doctors may be regarded as less experienced or less competent and perceived worse internationally - Logistical implications of increasing capacity (despite it being needed) should be considered. | - Graduates of fast track medical schools may find themselves trapped in the UK  - Challenges in implementation incl. increasing facilities and capacity for more med students, could impact med ed quality - are there enough doctors to do the training? - Issues with scarcity of training posts and risks of doctors in a state of career stagnation incapable of advancing leading to poor job dissatisfaction | Limited Degree Transferability / Risk of Two Tier Doctors, Placement Capacity Concerns, Educator/Academic Shortage |
| The NHS workforce plan | William L Palmer, Rebecca Rosen | 2023 | BMJ (British Medical Journal) | Opinion piece/Commentary/Editorial | N/A | N/A | N/A | N/A | UK | Yes | Generalised/All | - welcome and necessary step to solving the workforce challenges  - When it comes to training - too few people has shown us that it is best to err on the side of caution and minimise the risk of staff shortages (which is more costly than an oversupply) - It is good to have a home grown workforce  - Ramping up training will allow for additional places to be distributed in underserved areas of the country + can benefit push for more generalised staff.  - NHS leadership by introducing this plan will commit central gov. to an adequate clinical workforce (which is a success) - Lots of work has clearly gone into the plan, but there is a risk it joins the pile of previous redundant modelling exercises unless the initiative is taken soon for filling in the remaining policy gaps and delivering changes needed. | - unclear if it is funded (even the 2.4B) is not due until 2025 (after the general election) - Consequences of ramping up training is untested despite decrease in reduction of placement and increase in simulation  - plan does not explain how enough academic and clinical educators and supervisors can be found from a NHS workforce under pressure.  - no mention of the effect of lowering thresholds for accepting people onto clinical courses which would likely happen with increases in training numbers - Point above + new and shorter training courses leaves a lot of the problem solving to regulators. | Funding Lacking/Unclear, Educator/Academic Shortage, Placement Capacity Concerns |
| The risk assessment for NHS England's long term workforce plan should worry doctors | David Oliver | 2024 | BMJ (British Medical Journal) | Opinion piece/Commentary/Editorial | N/A | N/A | N/A | N/A | UK | Yes | Generalised/All | - Poor risk assessment of the LTWP - these included the timeline for delivery; the fact that the NHS service strategy was in development and so would change workforce needs - That “multiple layers of governance” involving multiple organisations might hamper planning and delivery | - Lack of adequate risk assessments the lack of impact assessment, and the failure to anticipate the backlash against some of the plan’s elements.  - There’s no cross-party commitment to the plan, which puts its delivery at risk after the next general election. - There’s currently no financial commitment and no detailed logistical delivery plan or timeline for delivering the aims of the workforce plan - For instance, is there any funding to pay for the workforce or medical and nursing school expansion or capital investment in facilities? | Unrealistic Expectations / Criticisms Ignored, Short-termism/Political Concerns, Funding Lacking/Unclear |
| Bridging accelerated medical programmes and workforce demands: a critical evaluation of the four-year direct entry medical degree | J. J. Lim, C. Roberts, R. Singh and J. Wellington | 2024 | Journal of the Royal Society of Medicine | Opinion piece/Commentary/Editorial/Analysis | N/A | N/A | N/A | N/A | UK | Not directly by name, but mentioned. | AUMDs | - Accelerated medical degrees were first introduced in the US during WWII to address physician shortages and over 30 medical schools in the US and Canada offer these shortened courses.  - Buckingham already has a 4.5 undergrad programme, which is manged by intensifying the schedule  - Drawing evidence from the US, accelerated medical programmes have proven to be multifaceted solution towards workforce demands, for example by recruiting students who want to work as GPs in remote areas, and by giving them specific training to help them meet these demands.  - These programmes will be slightly cheaper (but not entirely, because there still will need for additional resources like summer teaching - This 'can' further encourage applications to widen access - Current 3 year MD programmes in the US have excellent academic performance and a more positive learning environment, and lower levels of burnout compared to those in standard courses. - There should be ways to opt-in and opt-out of the accelerated programmes (due to the high number of people needing to extend their studies in the US).  - In developing 3-year MD programmes, there were ways in which sensemaking and establishing a community of practise involving stakeholders worked. - Complexity theory suggests curricular revisions should be guided by shared leadership and self-organisation  - A redoing of medical education requires multifaceted approach that not only tracks the graduates over time, but considers fiscal and social and educational goals. | - Accelerated medical programmes have been discontinued previously due to elimination of government funding and declining concerns over physician shortages and student dissatisfaction ( in the US). - Lack of formal engagement with relevant parties and lack of concreate evidence for its benefits. - Dilute medical education, ( time does not equal quality, but can a four year course match a five year in academic and clinical rigour?) - Financial pressures and competition for clinical placements remain unresolved - Unclear if a four year course will adequately prepare students for the MLA - Increased risk of burnout and mental health issues, For example in the US, 25% of students in accelerated MD programmes voluntarily extend their education due to high stress.  - Students may lose opportunities to develop essential soft skills, such as communication, leadership and empathy and the opportunities to engage in extracurricular activities - Worth noting that Buckingham: has a high proportion of international medical students as it does not have a 7.5% limit on international students like the other Uk universities; and with a fee-for-education model may have different admission criteria to other medical schools (especially having an impact on WP students) - this means that the relevance and transferability to other medical schools may be limited - May hinder WP students as they will be unable to work part time or manage caregiving responsibilities (further accentuating under-representation of students from disadvantages socioeconomic backgrounds).  - Curriculum reform requires financial and workforce investments. and medical schools with resource constraints may struggle to revise learning outcomes for a four year programme. it is more than simply compressing the existing five year model, but it will demand innovative re-engineering to balance MLA and clinical readiness. Leading to overburdened medical schools compromising on the quality of education. - Complexity theory suggests that changes in medical education are non linear and require flexible approaches with stakeholder feedback. | Student Burnout/Welfare Risk,  Funding Lacking/Unclear,  Lack of Stakeholder Engagement, Limited Degree Transferability / Risk of Two Tier Doctors, Risk to Widening Access, implementational Uncertainty / Lack of Detail |
| Written evidence submitted by The BMA to the Health and Social Care Committee Workforce report (RTR0070) | British Medical Association | 2022 | Health and Social Care Select Committee | (written) evidence | N/A | N/A | N/A | N/A | UK | Published before LTWP | Generalised/All | - 50,000 more FTE doctors would be needed to raise the doctor ratio in England to the average in the OECD.  - Overcoming unsafe staffing levels is an essential measure to ensure patient safety and boost wellbeing and morale. The need to publish regular independently verified assessments of the workforce are needed - BMA does not believe that the current outcome for graduates published by the GMC needs to be revised.  - BMA does not currently believe there is a safe substitute for the existing 5-year undergrad, or 4-year GEM courses, this could risk compromising their education and reducing the quality and comprehensiveness of patient care. - The number of medical students should be doubled and take advantage of the increases in medical student applicants. | - Lack of a detailed, long term workforce plan, built on data and modelling is an oversight which is needed to stabilise the current workforce crisis. - Pushing MDDAs forward would bring about fundamental changes to the way doctors are trained and are yet to be fully discussed in depth by the medical profession.  - Practically there will need to be a long-term strategy for the expansion of medical schools, to ensure there is enough teaching space, educators, and support for students on placement (and on the FP and specialty training). - Medical academic numbers have contracted by 27% despite a 25% growth in medical student numbers in the past 10 years. | Risk to patient safety, Lack of Stakeholder Engagement, Educator/Academic Shortage |
| RCP Responds to News That Revised NHS England Long Term Workforce Plan Will Be Published in 2025 | Royal College of Physicians | 2024 | Royal College of Physicians | Press Release | N/A | N/A | N/A | N/A | UK | Yes | Expansion of training places | - RCP welcomes confirmation of a LTWP refresh - A regularly refreshed plan with independently verified modelling is the right approach to ensure scrutinization of workforce proposals to meet patient demand - NAO recommendations that should be accepted into the plan, especially that assumptions should be generated in transparent and systematic consultation with external stakeholders - Right that this refresh comes after the publication of the 10 year plan, and current assumptions about staffing numbers, capacity and retention must feed into the plan - 10 YP must be ambitious but feasible  - UK government will need to fund the plan sufficiently, which will need to be reflected in the upcoming spending review. | - Set out more detail on expanding medical school places - Including plans for increasing educator capacity  - Plan should be generated in transparent and systematic consultation with external stakeholders  - Funding must be sufficient | Funding Lacking/Unclear,  Lack of Stakeholder Engagement,  Educator/Academic Shortage,  Implementational  Uncertainty / Lack of Detail |
